# Prevalence of Smokeless Tobacco among Low Socioeconomic Populations: A Cross-Sectional Analysis

**DOI:** 10.1371/journal.pone.0156887

**Published:** 2016-06-08

**Authors:** Mohammad Nurul Azam, Mohammad Shahjahan, Mahbuba Yeasmin, Nasar U. Ahmed

**Affiliations:** 1 King Saud University, Riyadh, Kingdom of Saudi Arabia; 2 Daffodil International University, Dhaka, Bangladesh; 3 Department of Public Health, Daffodil International University, Dhaka, Bangladesh; 4 EKRA Institute, Melbourne, Victoria, Australia; 5 Department of Epidemiology, Florida International University, Miami, United States of America; National Institute of Health, ITALY

## Abstract

**Background:**

Cost, social acceptability and non-stringent regulations pertaining to smokeless tobacco (SLT) product sales have made people choose and continue using SLT. If disaggregated data on smokeless forms and smoked practices of tobacco are reviewed, the incidence of SLT remains static. There is a strong positive correlation of SLT intake with the occurrence of adverse cardiovascular disease, particularly in the low socioeconomic populations.

**Aims:**

To investigate the prevalence of smokeless tobacco, its initiation influence and risk factors associated with the practice among lower socioeconomic populations of Bangladesh.

In this study, we explore the utilization of SLT among lower socioeconomic populations in industrialized zone of Bangladesh.

**Methods:**

A cross-sectional analysis using both quantitative and categorical approaches was employed. Using systematic random sampling method, four focus group discussions (FGDs) were conducted and 459 participants were interviewed. Multiple logistic regression model was applied to distinguish the significant factors among the SLT users.

**Results:**

Almost fifty percent of the respondents initiated SLT usage at the age of 15–24 years and another 22 percent respondents were smoking and using SLT concurrently. The bulk of the women respondents used SLT during their pregnancy. Nearly twenty five percent of the respondents tried to quit the practice of SLT and one-quarter had a plan to quit SLT in the future. More than twenty percent respondents were suffering from dental decay. A noteworthy correlation was found by gender (p<0.01), sufferings from SLT related disease (p<0.05). The multiple logistic regression analysis suggested that, males were 2.7 times more knowledgeable than that of females (p<0.01) about the adversative health condition of SLT usage. The respondents suffering from SLT related diseases were 3.7 times as more knowledgeable about the effect of the practice of SLT than the respondents without diseases (p<0.01). Regarding the knowledge about the health consequences of the practice of SLT, one participant in the FGD session commented that “*although the mouth is the gateway to health*, *we infected our mouth by using Zarda and Gul”*. Again, informants opined that peer, family, curiosity and hospitality, culture are influencing factors for SLT initiation.

**Conclusion:**

counselling on tobacco, including SLT, health hazards have to be emphasized through mass media and it is essential for development of relevant policies and communication messages to make people aware of serious health consequences of SLT usages.

## Introduction

Approximately 6 million people breathe their last every year as a consequence of use tobacco and SLT [1], it is the mainly representing about 12% global mortality of adult [2]. Keeping all conditions constant the SLT consumption and the forecasted death tool will be nearly 10 million every year by the time 2020 [3]. The SLT habit is a main factor of death that can be avoidable [4, 5]. Smokeless tobacco is in general health burden as the SLT epidemic is a greatest danger the world confronted ever. At present, utilization of tobacco makes 1 in 10 deaths among adults overall which trait above 5 million persons a year. Yearly fatalities from tobacco use will upsurge to additional 8 million by 2030, unless the critical move is made. In the event that present patterns hold on uncontrolled, it is estimated that around 500 million individuals active currently will be killed by tobacco. In this century, SLT may perhaps be the cause of deaths around a billion individuals [6]. This global agent of death kills up to half of its users, and this premature death of users deprive their families of income, raise the price of health care and hamper economic development comprehensively. Most ordinarily used SLT products incorporate—tobacco paan masala, tobacco with Chun (lime-calcium carbonate), and tobacco with pan and betel quid [7]. Customary structures like betel quid, tobacco with lime and tobacco tooth powder are usually used. SLT habit is the mainly preventable cause for unexpected loss and infection around the Earth, and this one consequence is significantly more affirmed in low-and middle-income countries [8]. South Asia is a noteworthy maker and a net exporter of tobacco. More than 33% of tobacco expended locally is smokeless. Tobacco leaf production has been expanding relentlessly and extensively, and has multiplied subsequent to 1960s [9]. The distribution of tobacco utilization is not even; it is excessively upper in lower socioeconomic clusters, poor and semi-gifted manual profession groups, unemployed and poor with no education.

The practice of SLT is rampant in South East Asia. According to World Health Organization (WHO), 90% of SLT consumers live in South East Asia [10]. In south Asia, including Bangladesh traditional values do not permit smoking by women, however, the usages of SLT is socially acceptable. Tobacco used with betel leaf, mostly used variety of chewing tobacco, known as Paan in the subcontinent, it is commonly used after having food, snacks, tea in small and large social gatherings, as a cultural practice of Bangladeshi people which is extending back over the centuries [11]. The prevalence of SLT propensity is 27% (26 million grown-up individuals) with a comparable circulation crosswise over sexual orientations in Bangladesh [12]. In Bangladesh, tobacco habit has become not only a major contributor to the country’s high morbidity, but also the biggest burden to the country’s economy [13]. Several nationwide research in Bangladesh revealed the high occurrence of both smoking (e.g., cigarettes, *bidis*) and habit of SLT (e.g., betel quid with Gul, tobacco, *khoini*, *Zorda*) [12].

Easy accessibility and affordability, along with misunderstandings concerning its useful health effects are main contributing elements for augmented smokeless tobacco consumption. While many people are aware that tobacco is hazardous, the majority of users are not aware about the lethal association between SLT and fatal diseases. The major health consequences related through SLT use include oral and dental diseases [14] which disproportionately affects the poor. Besides, it is linked with a 2–4 times increase in the risk for cardiovascular diseases.

The mortality hazard for ladies who expend SLT is greater than that for men. Utilization of SLT in pregnancy is connected to stillbirths and a 2–3 times higher danger of low conception weight babies. Other wellbeing impacts of SLT practice in corporate caries of tooth, subsiding of gums, hypertension, oral sub-mucous fibrosis (OSF), cancers of the mouth and food pipe etc. [10]. In Bangladesh is overburdened with tobacco-related illnesses; 57,000 people died in 2004 because of SLT related diseases. In one review by WHO, 9% of the members analyzed at families had no less than one of eight chose SLT related infections (ischemic coronary illness, lung growth, stroke, oral tumor, and malignancy of the larynx, endless obstructive pneumonic sickness, aspiratory tuberculosis or Buerger's ailment). Likewise, 41% of these were invariable from tobacco. The healing centre information showed that 29% of inpatients matured 30 years or above were hospitalized for the reason of infections.

It was also estimated that they were responsible for 16% of all demises in the country and 9% of the cases were due to tobacco [15]. Studies from India [7] and Pakistan [16] report that many people believe that roughly the SLT products are beneficial to health, relieving toothache, headache, and stomachache. Accessibility of SLT products is an main environmental factor that influences tobacco initiation [17, 18]. Generally, sun or air preserved SLT can be used by the person itself in unrefined, treated or factory-made form. It is used with lime, with Areca nut or in a betel quid (*pan*). The distribution of SLT intake is not uniform; it is excessively higher in lower socioeconomic clusters, poor, semi-skilled labor-intensive occupation clusters, unemployed and people with no education.

Bangladesh is one of the main manufacturers of tobacco in the world, SLT products is used by men, women, adolescent and children. New forms of highly addictive packaged SLT goods such as *Zorda*, *Gul*, *and Sadapatha* are cheap and rates of use are higher in low-income urban communities

So far we know no complete research on the initiation influence of the practice of SLT, the correlation between risk factors and the habit of SLT in Bangladesh. Due to lack of such data on SLT habit in Bangladesh, the aim of this study is to investigate the prevalence of smokeless tobacco, its initiation influence and risk factors associated with the practice among the low socio-economic populations of Bangladesh. This study covered two areas, viz. Demra and Tongi, adjacent to Dhaka Metropolitan city. These two adjacent areas of Dhaka were selected for the study as these are industrial areas and easily approachable. Traditionally, this cohort of population use smokeless tobacco higher than others (Gilani et al., 2013). Usually in industrial areas, low income people live and supposed to use smokeless tobacco more frequently than others.

## Materials and Methods

### Study Design and Study Sites

This Cross-sectional research was piloted in two areas, viz. Demra and Tongi, adjacent to Dhaka Metropolitan city of Bangladesh during March-November, 2014. These two adjacent areas of Dhaka city were chosen by simple random sampling for the research as these are industrial areas may represent the country as whole and easily approachable to the research team. Usually in Industrial areas, low income people live and supposed to habit of SLT more often than others. Traditionally, this cohort of population use SLT higher than others [19].

### Study Population

People lived in Demra and Tongi industrial areas those habits of SLT and meet the following inclusion, along with exclusion criteria were chosen for this study.

### Sample Size

The study respondents were all SLT users. A total of 460 consecutive eligible SLT users aged 15 years and above were recruited from both Demra and Tongi areas. Together a male and female participations from two areas were considered.

The sample size was determined by using the following formula.

$$n=z^{2}pq/d^{2}\times (\text{design effect});$$

Where, *n* = desired sample size, *z* = 1.96 (at 95% confidence interval), *p* = Prevalence of smokeless tobacco = 27.2% [20], *q* = 1−*p*, *d* = precision level, Design effect = 1.5, = (1.96)^2^ (0.272) (0.728)/ (0.05)^2^[× design effect], = 304.3 × design effect = 460 (Approximately).

### Sampling Technique

A detail investigation was accompanied prior to actual data gathering to classify the objective residents by sex. Enumeration checklist was developed to list entitled household participants. The checklist contained Line number, Name of the person, Relationship with household head, Gender, Education, Habit of SLT habit and Number of SLT users in the family. A sampling frame was constituted with this information stratified by sex. A systematic sampling technique was applied to get the target sample from this sampling frame.

### Data Collection Approach

Using semi-structured questionnaire information were collected at the household level. The questionnaire was pre-tested in a non-sample site in an industrial area within Dhaka city with a draft Bangla version of the instruments to get feedback on the suitability, appropriateness and sequencing of the questions. The study included 230 from Demra (115 males and 115 females) and 230from Tongi (115 males and 115 females) respectively for interview. The face-to-face interview technique was applied to collect data from the SLT users. For each eligible respondent’s a questionnaire including socio-demographic data for example Age, gender, level of education, marital status, occupation, Family History of SLT, Income, religion, Living area, Family members, Numbers of Members used SLT, Mobile use was completed. Information about SLT use, influence on the start of SLT habit and related risk elements were collected.

### Inclusion and Exclusion Criteria

People lived in Demra and Tongi industrial areas those uses smokeless tobacco and meet the following inclusion as well as exclusion criteria were chosen for this study. Inclusion criteria: Smokeless tobacco user (with or without smoking habit), age 15 years and above, both male and female, physically able and willing to participate. Exclusion criterion: very sick or very old person, temporary migrant (guest)

### Data analysis plan

The collected information was checked for errors, coded and pass in into a database using SPSS software. Analysis targeted the research objectives by considering the indicators. Descriptive analysis of all relevant variables was done using measures of central tendency, Association and differentials within/between variables was tested using appropriate test. Statistical tests used to regulate the relationship between exposure and outcome variables included χ2 test, Fisher’s exact test, independent sample t-test and ANOVA. To test the statistical significance, the p value level of <0.05 was considered. Logistic regression was applied to find out the factors influencing health effects due to SLT uses among low income peoples. Special effects of exposure variables were similarly measured once correcting for added variables by multivariate analyses. In addition to Odds Ratio (OR), 5% level of significance of different estimates was also estimated.

### Ethical Issues

Before starting the data collection, the interviewers briefed the respondents about the objectives of the research to make him/her mentally ready for specific question. An informed written consent was signed from the respondents before the interview and was ensured not to disclose their personal information. The study received approval from the ethical agency of Bangladesh Medical Research Council (BMRC).

### Operational Definitions

Low socioeconomic populations: The household income, expenditure survey 2010 of BBS suggested that TK. 1250 per person per month is categorized as the poor living in low socioeconomic areas at the nationwide that translates to an average income of TK. 5500 per family per month.

Low Income people in the industrial belt: As per the definition of BBS, per capita earning of Taka 1250 or less is considered as low income group people [21]. It would be far higher in line for to inflation and other factors over time. However, in the industrial areas in the periphery of the capital, scope of earning of such group of people is much greater than other areas of the country. It was observed that the income of respondents is about 46 percent higher than that of the reference cutoff point.

Industrial Area: The areas where usually employees of industries along with their relatives reside. In the proposed study, Tongi and Demra are considered as Industrial areas. There are Pharmaceuticals, Garments, Steel and Re-rolling and many other Industries. Essential commodities of SLT were produced in these areas.

Demarcation of the study zone: Firsthand information was gathered by visiting the spots of the research zone. Two adjacent zones of Dhaka Metropolitan city, viz. Tongi and Demra, were selected for the research as these are industrial areas, easily approachable and traditionally they practice SLT higher than others.

## Results

### Socio-demographic features of the participants

Details of socio-demographic features of the participants are portrayed in Table 1.

**Table 1:** Socio-demographic features of survey participants.

Background variable	Male n (%)	Female n (%)	P-value
Age (years)			
15–25	6 (2.6)	7 (3.0)	0.45
26–35	58 (25.2)	56 (24.3)	
36–45	94 (40.9)	91 (39.6)	
46–55	47 (20.4)	37 (16.2)	
56–65	17 (7.4)	29 (12.6)	
66+	8 (3.5)	10 (4.3)	
Mean±SD	42.53±11.12	43.97±11.24	
Area			
Demra	115 (50.0)	115 (50.0)	0.99
Tongi	115 (50.0)	115 (50.0)	
Educational status			
No formal education	115 (50.0)	151 (65.7)	<0.001
Primary	36 (15.7)	42 (18.2)	
Secondary	63 (27.3)	34 (14.8)	
HSC and above	16 (7.0)	3 (1.3)	
Occupation			
Housewife	1 (0.4)	142 (61.7)	<0.001
Service	92 (40.0)	11 (4.8)	
Business	88 (38.3)	10 (4.3)	
Garments workers/Day labourer/Others	49 (21.3)	67 (29.2)	
Marital status			
Married	222 (96.5)	190 (82.6)	<0.001
Unmarried/Divorced/Widow	8 (3.5)	40 (17.4)	
Family size			
≤4	132 (57.4)	117 (50.9)	0.16
> = 5	98 (42.6)	113 (49.1)	
Family history of use of SLT			
Yes	83 (36.1)	63 (27.4)	0.045
No	147 (63.9)	167 (72.6)	

The findings were presented as n (%) and mean±SD, χ2-test was used. *The level of significance at α = 0.05, (The P-values in the last column are the significance level of the Chi-square distribution for each 2-way contingency tables)

A total of 230 female and 230 male respondents was included in this study. More than one-third of respondents belong to 36–45 years age group. The mean age of man and woman were 42.53± 11.12 vs. 43.97±11.24 years respectively. An equal proportion of men and women were sampled from Demra and Tongi area. A positive correlation was found regarding other socio-demographic features of the male and female, such as educational status (p<0.001), occupation (p<0.001), marital status (p<0.001) and the family record of SLT habits (p<0.045).

### Behavioral characteristics of SLT user by gender

Approximately 7% of the adult males and eight percent of the female initiated SLT habits below the age of 15 years (Table 2).

**Table 2:** Features of smokeless tobacco consumers by sex.

Background variable	Male n (%)	Female n (%)	p-value
Age of initiation (years)			
≤15	15 (6.5)	18 (7.8)	0.06
15–24	88 (38.3)	110 (47.8)	
25–34	96 (41.7)	67 (29.1)	
35–44	23 (10.0)	22 (9.6)	
45+	8 (3.5)	13 (5.7)	
Mean±SD	26.43±8.57	25.83±9.75	0.48
Types of use of SLT*			
Zarda	216 (98.2)	203 (91.9)	<0.001
Sadapata	7 (3.2)	23 (10.4)	
Gul	13 (5.9)	22 (10.0)	
Panmasla	6 (2.7)	8 (3.6)	
Number of use of SLT/day			
< = 5	70 (30.4)	103 (44.8)	<0.005
6 to 10	101 (43.9)	89 (38.7)	
11 to 15	32 (13.9)	15 (6.5)	
16–20	12 (5.3)	14 (6.1)	
20+	15 (6.5)	9 (3.9)	
Mean±SD	10.05±08.0	08.34±6.67	0.013
Duration of use of SLT (years)			
1–5	37 (16.1)	41 (17.8)	0.16
6–10	43 (18.7)	37 (16.1)	
11–15	46 (20.0)	32 (13.9)	
16–20	45 (19.6)	40 (17.4)	
≥21	59 (25.6)	80 (34.8)	
Mean±SD	16.10±10.28	18.13±11.91	0.05
Currently using cigarette, bidi along with SLT			
Yes	101 (43.9)	3 (1.3)	<0.001
No	129 (56.1)	227 (98.7)	
Number of cigarette smoked per day**			
Up to 10	55 (54.5)	2 (66.7)	0.99
More than 10	45 (45.5)	1 (33.3)	
Mean±SD	14.31±9.79	10.00±8.87	0.45

The findings were presented as n (%) and mean±SD, χ2-test and t-test were used. *The level of significance at α = 0.05

*Using the Multiple Response Sets option of the table’s procedure.

**Using Fisher’s exact test. The P-values in the last column are the significance level of the Chi-square distribution for each 2-way contingency tables)

Approximately 50% female users initiated at the age of 15–24 years and about 42% of males initiated at age 25–34 years. The mean age of beginning of SLT habit was found to be 26.43±8.57 years in males and 25.83±9.75 years in females respectively. In this research the respondents mostly use *zarda* than other SLT. Around 98 percent of males use *zarda*, while it is 92 percent among women as observed. Use of *sadapata* and *Gul* was found higher among females (10.4 percent and 10.0 percent, respectively) in comparison to males (3.2 percent and 5.3%, respectively). The types of SLT habit (p<0.001), number of SLT practices per day (p<0.005) and currently using cigarettes, *the bidi* along with SLT (p<0.001) were positively correlated among man and woman users. In independent sample t-test, number of SLT intake per day was found significantly higher (p<0.013) in Male (10.05±08.0 per day) than Female (8.34±6.67per day). On the other hand, the mean time period of SLT practice was significantly higher (p<0.05) in female (18.13±11.91 years) than that of males (16.10±10.28 years).

### Factors on frequency of SLT practice and its association with diseases

The respondents took SLT on average 9.0 times per day regularly but there was variation by area, gender, educational status and occupation (Table 3).

**Table 3:** Frequency of smokeless tobacco daily use and social-demographic features.

Characteristics	N	Mean	SD	p-value	Adjusted for Socio-demographic variables		
					β	F-value	p-value
Area							
Demra*	230	11.57	9.1	<0.001			
Tongi	230	6.83	3.99		-0.32	52.3	<0.001
Gender							
Male	230	10.05	8	0.013	0.12	6.2	0.013
Female*	230	8.34	6.67				
Age (years)							
≤25*	13	5.23	2.39	0.407			
26–35	114	9.81	9.34		0.267		
36–45	185	9.16	6.75		0.26		
46–55	84	8.99	7.43		0.196		
56–65	46	8.87	5.46		0.147		
66+	18	10.33	6.35		0.134	1.016	0.407
Educational status							
No formal education*	266	9.15	6.84	0.047			
Primary	78	8.1	7.01		-0.053		
Secondary	97	10.69	9.3		0.085		
HSC and above	19	6.63	4.07		-0.068	2.672	0.047
Occupation							
Housewife*	143	9.26	7.41	0.029			
Service	103	9.5	7.65		0.013		
Business	98	10.63	8.45		0.076		
Garments workers/ Day laborer/Others	116	7.64	5.9		-0.095	3.037	0.029

*Reference group, p-value by t-test, one-way ANOVA

Respondents from Demra (p<0.001) and Males (p<0.013) used SLT significantly more than their colleagues. The frequency of SLT practice augmented with age and peaking in the 66 and above year age group. SLT practice was found significantly more among the respondents who have secondary education than others (p<0.047). Businessmen used it significantly more often than others (the housewife, servicemen and garments workers or day laborer) (p<0.029). Multiple regression models showed an insignificant effect of age on the frequency of SLT use after controlling for other variables (Table 3).

Table 4 shows the logistic regression analysis considering the respondent suffering from any of the selected diseases as the response variable and area, age, gender, educational status, occupation, frequency of SLT practice and periods of SLT practice as independent variable.

**Table 4:** Results of logistic regression with smokeless tobacco habit related disease.

Independent variables	β*	p-value	OR**	95% CI for Exp (β)	
				Lower	Upper
Area					
Demra	Reference				
Tongi	0.093	0.709	1.097	0.675	1.783
Gender					
Female	Reference				
Male	0.253	0.472	1.288	0.646	2.567
Age (years)	0.022	0.105	1.022	0.995	1.05
Educational status					
No formal education	Reference				
Primary	0.014	0.964	1.014	0.542	1.898
Secondary	0.135	0.644	1.145	0.645	2.034
HSC and above	1.051	0.039	2.86	1.053	7.764
Occupation					
Housewife	Reference				
Service	0.417	0.35	1.518	0.633	3.638
Business	0.506	0.246	1.659	0.706	3.897
Garments workers/ Day labourer/Others	-0.028	0.94	0.972	0.467	2.022
Frequency of use of SLT times/day					
<5	Reference				
6–10	0.513	0.054	1.67	0.992	2.814
11–15	0.07	0.869	1.073	0.465	2.474
16–20	0.361	0.481	1.434	0.526	3.914
21+	1.194	0.015	3.301	1.263	8.628
Duration of use of SLT	0.004	0.794	1.004	0.977	1.031
Constant	-2.851	0	0.058		

* Coefficient

** Odds Ratio

The analysis suggests that male were more likely to suffer from the selected diseases than females (OR 1.288; 95% CI: 0.646 to 2.567) however, it did not achieve statistical significance. Suffering from any disease increased significantly with the less frequently SLT use to more frequently SLT use (p<0.015; OR 3.301; CI: 1.263 to 8.628).

## Discussion

Since tobacco use has been conveyed to be greater among the poor and less educated people, both disease burden along with an economic burden due to tobacco use will disproportionately affect them [22]. SLT use is a habit with adverse consequences ranging from benign oral lesions to cancer. The practice of the SLT increase in all countries from highly industrialized ones like Sweden and Canada to developing ones like Nepal and Bangladesh [23].

The mean age of instigation of SLT practice was found to be 26.4±8.6 years in males and 25.8±9.8 years in females respectively. The frequency of SLT practice increased with age highest in the 66 and above year age group. Another study in Bangladesh mentioned that the mean age at the onset of SLT practice was 31.5 years [24]. This was more than our study findings, our sample came from low-income industrial areas. It is expected that low-income people started using SLT earlier as it is socially acceptable, less expensive entertainment [25]. It reveals that the frequency of SLT practice increases with age is dependable with previous reports [12, 13, 26]. This increased likelihood of SLT practice is correlated to the social acceptance of SLT consumption by older people and a greater appeal of cigarette among the younger generations who may be taking up smoking instead of SLT consumption. The average time of SLT practice was significantly higher in female (18.13±11.91 years) than that of males (16.10±10.28 years). Prior start would be required to build the infection load by expanding clients' duration of lifetime exposure to carcinogens [24].

In this study the most frequent form of SLT intake was zarda than other SLT. Around 98 percent of males use zarda, while it is 92 percent amongst females as observed. These findings are higher than other Bangladeshi studies [27, 14]. Use of sadapata and gul was higher among females (10.4 percent and 10 percent, respectively) in comparison to that of males (3.2 percent and 5.3 percent respectively). But no significant gender differentials were witnessed in the occurrence of gul use in other studies in Bangladesh [28]. It is revealed from the data that gul use is (10 percent among female and 5.3% among male) greater than the preceding Bangladeshi study [28]. A USA study found that gender is highly related to SLT users (4 percent), where males found the largest group of SLT users with the frequency rate of 8 percent, while among females it was near to 1 percent [29].

This study revealed that the brands of SLT practice (p<0.001), number of SLT intake per day (p<0.005), mean length of SLT practice (p<0.05) and currently using a cigarette, bidi along with SLT (p<0.001) were positively correlated among man and woman users. Several factors influence SLT use, including ethnicity, demographic and psychosocial factors, its accessibility and public policy. Personal habits are dictated by different communal and traditional behaviors in both sexes [30].

SLT habit was found meaningfully more recurrent among the respondents who were educated up to secondary level than others (p<0.047). The expanded likelihood of SLT use among the ignorant and less taught Bangladeshis might show that comparable variable are included in the start of SLT utilize and smoked tobacco among Bangladeshis [12, 13, 31]. Comparable financial contrasts in SLT use were additionally reported in India [26, 32]. Education always plays a vigorous role in the health status of a country and also in the progress of healthy behavior of a person. Our study also showed that business persons, housewives, servicemen and garment workers used SLT frequently. These occupational groups are maximum at hazard for the exposure of SLT usage than other occupations.

According to WHO, SLT practice is the main cause of mouth cancer. Due to the high use rate of SLT habit, South East Asia carries the highest burden of mouth cancer as over 95,000 cases each year suffer from it; 50% are caused by tobacco [8]. Oral cancer affects the poor class [8], who has a greater coverage to SLT and is consistent with the current study findings. SLT usage is the crucial risk element for non-communicable and communicable diseases. Logistic regression analysis suggests that male were more likely to suffer from SLT related diseases than females (OR 1.288; 95% CI: 0.646 to 2.567). Suffering from any disease increased significantly with the less frequently SLT use to more frequently SLT use (p<0.015; OR 3.301; CI: 1.263 to 8.628). A study in Dhaka concluded that tobacco consumption-either through mastication or smoldering–was a vital aspect in the growth of mouth cancer [33]. Studies conducted in Asia exhibited that the mortality danger for females who ingest SLT is larger than that for men [8]. The issues associated with the SLT habit in this paper found in a survey in India [32]. The practice of SLT practice is almost alike in the low educated population groups in India also in Sri Lanka [32]. Low level of education constitutes as a significant indicator of the consumption of SLT, another factor is wealthy family for both man and woman [34]. It is because the low education status of the people is less alert of the health risks of the practice of SLT and have a bigger chance to have a fatality and risk [35].

## Conclusion

The practice of SLT start is simple as it is worthy and affected by the general public, family and companions. The SLT consumption was witnessed to be a typical practice in Bangladesh. Experienced grownups (26+years) of low socioeconomic status, living zone, occupation and educational status will probably be connected with the use of SLT. Anyone, irrespective of age can easily buy every single SLT item. The absence of information on the leaving strategy, structure identified by smokeless type of tobacco and is almost a neglected policy area. In Bangladesh there is no public policy related to SLT. The outcomes of this study could be considered for defining future SLT control approaches in Bangladesh. The products of the SLT are cheap and simply reachable to the users in Bangladesh. We found that those who practice SLT are unequivocally connected to the users of low financial status and less education. An extensive embargo on SLT use should be executed by the policy makers established in the standard guideline in 'Article 13' in the Charter Agreement of WHO [36]. In addition a requirement for an across the nation campaign instructing individuals about the wellbeing danger of SLT consumer.

### Ethical Issues

Before initiating the data collection, the interviewers briefed the participants about the objectives of this study to make them mentally ready for specific question. Up-to-date written agreement was requested from each data collector in the prescribed consent form. The partakers earlier the meeting was guaranteed not to uncover their own data. Security and secrecy were kept up in regards to the gathered data. The convention, including the data sheet and consent forms for this study was approved from Bangladesh Medical Research Council (BMRC) ethical committee.

## Supporting Information

S1 File S1 SLT Data data used in this study. (XLSX)
